# Functional Repetitive Neuromuscular Magnetic Stimulation (frNMS) Targeting the Tibialis Anterior Muscle in Children with Upper Motor Neuron Syndrome: A Feasibility Study

**DOI:** 10.3390/children10101584

**Published:** 2023-09-22

**Authors:** Leonie Grosse, Anne C. Meuche, Barbara Parzefall, Corinna Börner, Julian F. Schnabel, Malina A. Späh, Pia Klug, Nico Sollmann, Luisa Klich, Matthias Hösl, Florian Heinen, Steffen Berweck, Sebastian A. Schröder, Michaela V. Bonfert

**Affiliations:** 1 Division of Pediatric Neurology and Developmental Medicine, Department of Pediatrics—Dr. von Hauner Children’s Hospital, LMU Hospital, Ludwig-Maximilians-Universität München, 80336 Munich, Germany; 2 LMU Center for Children with Medical Complexity—iSPZ Hauner, Ludwig-Maximilians-Universität München, 80336 Munich, Germany; 3 Department of Diagnostic and Interventional Neuroradiology, School of Medicine, Klinikum rechts der Isar, Technical University of Munich, 81675 Munich, Germany; 4 TUM-Neuroimaging Center, Klinikum rechts der Isar, Technical University of Munich, 81675 Munich, Germany; 5 Department of Diagnostic and Interventional Radiology, University Hospital Ulm, 89081 Ulm, Germany; 6 Specialist Center for Pediatric Neurology, Neurorehabilitation and Epileptology, Schoen Clinic Vogtareuth, 83569 Vogtareuth, Germany; 7 Gait and Motion Analysis Laboratory, Schoen Clinic Vogtareuth, Krankenhausstr. 20, 83569 Vogtareuth, Germany

**Keywords:** repetitive peripheral magnetic stimulation, neurostimulation, neuromodulation, cerebral palsy, hemiparesis

## Abstract

Non-invasive neurostimulation as an adjunctive intervention to task-specific motor training is an approach to foster motor performance in patients affected by upper motor neuron syndrome (UMNS). Here, we present first-line data of repetitive neuromuscular magnetic stimulation (rNMS) in combination with personalized task-specific physical exercises targeting the tibialis anterior muscle to improve ankle dorsiflexion (functional rNMS (frNMS)). The main objective of this pilot study was to assess the feasibility in terms of adherence to frNMS, safety and practicability of frNMS, and satisfaction with frNMS. First, during 10 training sessions, only physical exercises were performed (study period (SP) A). After a 1 week break, frNMS was delivered during 10 sessions (SPC). Twelve children affected by UMNS (mean age 8.9 ± 1.6 years) adhered to 93% (SPA) and 94% (SPC) of the sessions, and omittance was not related to the intervention itself in any case. frNMS was safe (no AEs reported in 88% of sessions, no AE-related discontinuation). The practicability of and satisfaction with frNMS were high. Patient/caregiver-reported outcomes revealed meaningful benefits on the individual level. The strength of the ankle dorsiflexors (MRC score) clinically meaningfully increased in four participants as spasticity of ankle plantar flexors (Tardieu scores) decreased in four participants after SPC. frNMS was experienced as a feasible intervention for children affected by UMNS. Together with the beneficial effects achieved on the individual level in some participants, this first study supports further real-world, large-scale, sham-controlled investigations to investigate the specific effects and distinct mechanisms of action of frNMS.

## 1. Introduction

Congenital or acquired brain injury is the main cause of physical disability in childhood [1,2,3,4,5]. The common pathophysiological mechanisms underlying the motor dysfunction are summarized within the concept of the upper motor neuron syndrome (UMNS). The individual picture of motor impairment depends on the etiology and location as well as the size of the lesion(s), as well on the time point of injury during the lifespan (e.g., pre-, peri-, or postnatal). Specifically, the clinical picture is (1) classified as unilateral (hemiparesis) or bilateral; (2) categorized as a spastic, dystonic, or ataxic type; and (3) assigned as cerebral palsy in case of pre-, peri-, postnatal, or very early childhood injury or movement disorder if acquired later during childhood. Children with UMNS experience motor dysfunction due to muscular spasticity on the one hand and, to the same extent, due to muscular weakness and impaired selective motor control on the other hand.

Toe standing and walking due to overactivity/spasticity of the triceps surae muscle is one of the most common clinical symptoms in children with UMNS. Weakness and impaired selective motor control of the tibialis anterior muscle contribute to the pathologic and inefficient gait pattern by limiting plantar dorsiflexion during the swing phase while walking [2,6,7]. This may be compensated by a circumduction or supination of the foot but may also cause tripping, stumbling, and falls, in particular in situations of shared attention, uneven grounds, or higher walking speeds [8]. If these muscular imbalances are not adequately addressed, contractures of the plantar flexors can exaggerate the impairment during standing and walking. Lastly, secondary neuro-orthopedic malalignments of the ankle joints often associated with pain are likely to arise if misloading continuously occurs [7,9,10,11].

According to the framework of the International Classification of Functioning, Disability, and Health—Children and Youth Version (ICF-CY), UMNS results not only in limitations at the level of body function and structure but also activity and participation [12]. Clinical management of UMNS requires a multimodal, interdisciplinary approach tailored to the individual needs of each patient [13,14].

Currently, in high-income countries, established spasticity management includes physical exercise, orthoses, and/or the intramuscular injection of botulinum toxin [15,16]. Furthermore, goal-directed and task-specific motor training, mobility, fitness, and strength training as well as treadmill training represent the most important approaches to enhancing power and endurance during standing and walking [16,17,18,19]. However, the efficacy of these motor interventions is likely to be limited in children, who are unable to selectively control a distinct muscle or muscle group.

Non-invasive neurostimulation as an adjunctive intervention to task-specific motor training is a non-pharmacological approach to foster motor performance in this situation [20,21,22,23,24]. In this context, repetitive neuromuscular magnetic stimulation (rNMS) is a bottom-up approach based on the principle of electromagnetic induction. The magnetic stimulation induces a physiological-sized electrical current within the stimulated tissue. This current provokes a muscular contraction by activating the local terminal motor branches [25,26,27]. This externally evoked, physiologically dimensioned muscle contraction facilitates functional training aimed at strengthening the muscle as it overcomes the issue of impaired selective motor control. On the local muscular level, the repetitive contractions are hypothesized to induce the same mechanisms as voluntarily controlled concentric muscular training in healthy persons. Here, mechanisms involved related to an increase in strength are, e.g., an increase in cross-section and volume, an increase in fascicle length and pennation angle, an increase in fiber contractility and differentiation, an increase in blood flow, and, last but not least, an improvement in cellular and muscular metabolism [28,29,30,31,32,33,34,35,36,37].

Beyond having a local, direct muscular effect, rNMS massively increases the inflow of sensory information to the central nervous system both indirectly, by activating muscle spindles and mechanoreceptors in the contracting muscle–tendon units, the joints, and the skin, and directly, by depolarizing terminal afferent nerve branches [38]. These mechanisms of action modulate cortico-spinal excitability, affect central sensorimotor processing, and aim at inducing mechanisms of network reorganization and reactivation [39,40]. In previous work, other research groups have referred to rNMS as repetitive peripheral magnetic stimulation (rPMS) [20,21,25,39,40]. Our research group decided to introduce the term repetitive neuromuscular magnetic stimulation, as we have the impression that this term reflects the above-described biological mechanisms of the approach more comprehensively.

Transcutaneous or neuromuscular electrical stimulation (TENS and NMES) represent alternative neurostimulation approaches. The advantages of rNMS over such electrical stimulation are that it is painless, there is no need to attach electrodes or cables or to take off clothes, and it has the potential to efficiently reach deeper located and larger muscles due to the physical properties of the magnetic field [20,21,41].

Given the concept of windows of opportunity to achieve sustainable motor improvements, novel, non-pharmacological, non-invasive, and safe treatment options are highly needed for the vulnerable group of children affected by UMNS [42,43,44,45]. To keep the children on track during an intervention, it is highly important that the treatment feels comfortable, is easily applicable, and takes place in a way and setting that motivates the children to focus during the session and adhere to the planned training schedule. As rNMS is a non-invasive, painless treatment option, we hypothesized that it would be especially suitable for children. Next, it can be speculated that the effects of a neuromodulating treatment approach like frNMS has an even more pronounced effect on the developing brain of children than in the brain of adults [20,21,38,39,40,42].

Against this background, our research group developed an intervention comprising 10 sessions of rNMS in combination with simultaneously performed personalized, task-specific, physical exercises targeting the tibialis anterior muscle (functional rNMS (frNMS)).

The frNMS intervention was designed to empower ankle dorsiflexor function in children affected by bilateral or unilateral UMNS. By specifically targeting the frNMS to this muscle, we aimed to improve motor function (active dorsiflexion) and body structure (lower extent of plantarflexion during the swing phase, less risk for secondary neuro-orthopedic malalignments) and address activity and participation by improving the mobility (less tripping, stumbling, and falling and a more efficient gait pattern) of the children.

The primary aim of this pilot study was to assess the feasibility in terms of adherence to frNMS, safety and practicability of frNMS, and satisfaction with the frNMS protocol in children with UMNS. We hypothesized the adherence rate to be high (≥90%). In addition, the following clinical and patient-reported outcomes were preliminarily explored, hypothesizing beneficial effects of frNMS: participant/parent reported outcome, strength of ankle dorsiflexors, plantar dorsiflexors’ spasticity, and performance in the 10 m walking test (10 MWT).

## 2. Materials and Methods

### 2.1. Ethical Approval

Ethical approval was obtained from the internal review board of the Medical Faculty (vote 19-904). The study was registered in the German Registry for Clinical Studies [46]. The study was conducted in accordance with the Declaration of Helsinki. Informed written consent of participants and their caregivers was a prerequisite for study participation.

### 2.2. Study Design

**Figure 1:**
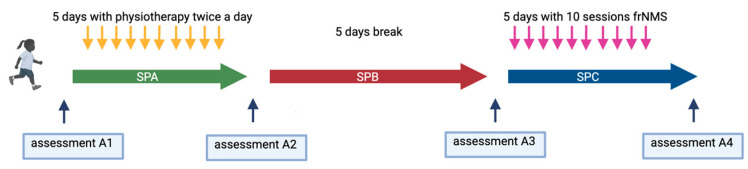
Study design of the single-center, prospective, intra-subject controlled, open-label clinical pilot study including 12 participants aged 6 to 11 years (created with BioRender.com).

We conducted a single-center, prospective, intra-subject controlled, open-label clinical pilot study. The study included a sequence of 3 study periods (SPs), each lasting for 5 days: SPA physiotherapy, SPB break without specific training, and SPC frNMS intervention (Figure 1). Clinical assessments (As) were completed before SPA (A1) and after SPA (A2) as well as before SPC (A3) and after SPC (A4). During SPA, all participants underwent physiotherapy 2 times a day, adding up altogether to 10 sessions comprising physical exercises focusing on strengthening the tibialis anterior muscle based on the concept of promoting motor learning [47]. During SPC, 10 sessions of frNMS targeting the tibialis anterior muscle took place. This study design was chosen since data from rehabilitation and training research report significant local effects on the muscular level after 9 to 10 conventional physical training sessions, and previous work by Flamand et al. demonstrated beneficial effects of a static rNMS treatment comprising 5 sessions of stimulation [30,34,36,48,49].

### 2.3. Study Population

Patients with UMNS in the context of unilateral or bilateral cerebral palsy who were admitted for inpatient neurorehabilitation were screened for study eligibility. The inclusion criteria comprised a Gross Motor Function Classification System (GMFCS) level of I to III, age between 6 and 12 years, and foot drop due to weakness of the dorsiflexors (muscle power value < 4 according to the Medical Research Council (MRC) scale) [50,51]. The exclusion criteria covered contraindications for magnetic stimulation (i.e., epilepsy, ferromagnetic implants, and implanted biomedical devices, including shunts), intellectual disability (IQ < 70), confirmed attention deficit (hyperactivity) disorder, and orthopedic surgery on or injection of botulinum toxin in the lower limbs within the previous 3 months. If the patient was eligible for the study, the patient and caregivers were informed about the course of the study and were offered participation.

### 2.4. Intervention

Physiotherapy: During SPA, participants received 10 sessions of task-specific training by board-certified physiotherapists [16]. The sessions took place twice a day and lasted about 45 min with 15 min of net training time. The participants performed 3 exercises from a previously developed catalog of exercises. This catalog included 16 different exercises grouped into 3 categories: static (the participant only moved the foot), activating (the participant completed a different task, such as playing with a ball, while performing the exercise), and dynamic (whole-body exercise, including movement demanding the activation of the tibialis anterior muscle; Table 1). All exercises were customized to the participant’s individual abilities and applied in a child-friendly setting. One exercise from each category was chosen by the therapist and the participant for each session. Different exercises were used throughout the intervention period to prevent habituation effects. All physical exercises were performed against gravity/using the participant’s body weight without any additional load. This approach as well as training applying distinct anti-gravity/(partial) body weight supported measures are well-established settings for motor interventions in pediatric cerebral palsy [52,53].

**Figure 2:**
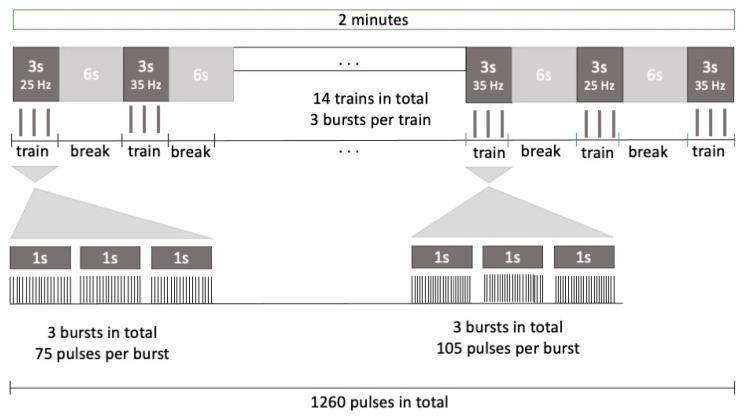
Stimulation protocol to target the tibialis anterior muscle during the frNMS intervention.

frNMS: During SPC, 10 sessions of frNMS targeting the tibialis anterior muscle took place. Within the functional approach, the participant performed the same physiotherapeutic exercises as during the respective session of SPA while frNMS was applied to the tibialis anterior muscle of the (more) affected lower limb. Each session also lasted about 45 min, including a net stimulation training time of 15 min. The stimulator (emFieldPro, Zimmer MedizinSysteme GmbH, Neu-Ulm, Germany) was equipped with a round coil delivering a maximum output of 2.5 Tesla. The coil’s copper winding had a 7.6 cm diameter, and the coil was equipped with an oil-based self-cooling system. Stimulation was delivered by emitting pulses of a rectangular shape for a duration of 250 µs with the direction of the induced current from the outside to the inside of the coil. Stimulation consisted of a total of 9450 pulses with alternating frequencies of 25 and 35 Hz, with 3 s of ON-time and 6 s of OFF-time and 15 min of net stimulation time. This resulted in 60 trains, including 3 bursts per train and 25 or 35 pulses per burst (75 or 105 pulses per train, Figure 2). These specific stimulation parameters were chosen because previous studies have reported neurophysiological cortical effects after rNMS with the following settings: frequencies higher than 10 Hz, at least 6000 total stimuli applied during 1 session, and at least 15 min of net stimulation time [54,55,56]. The alternation of frequencies was chosen to prevent habituation to the stimulation. From our own experience and in line with the biological properties of muscular tissue, higher stimulation frequencies are associated with discomfort, while lower frequencies are likely to only provoke singular twitches instead of a proper muscle contraction.

**Figure 3:**
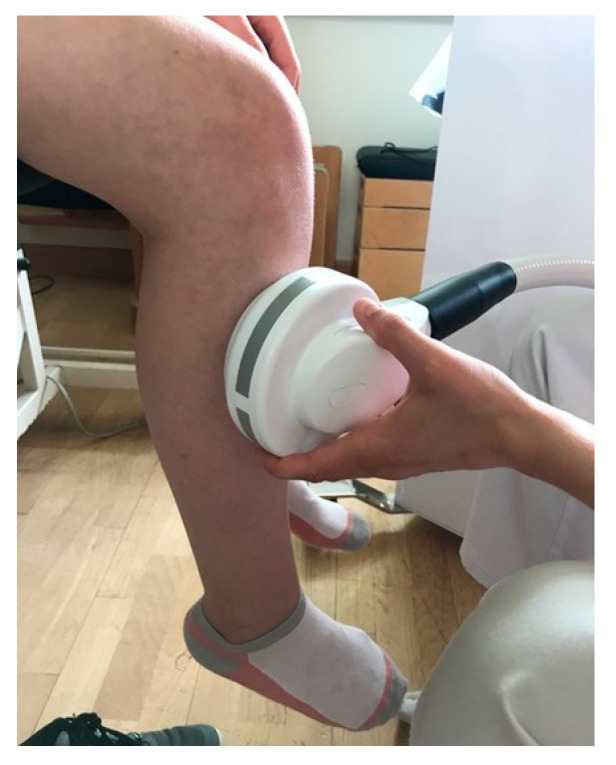
Position of coil held by therapist (upper third of the lateral lower leg) in the position that assured the most appropriate contraction of the tibialis anterior muscle.

During stimulation, the coil was held in the hand by the therapist and placed onto the upper third of the lateral lower leg in the position that assured the most appropriate contraction of the tibialis anterior muscle (Figure 3). The optimal coil positioning and stimulation intensity (percentage of maximum stimulator output) were repeatedly sought for each subject and session; starting at 20% of the maximum stimulator output, the intensity was usually slowly increased in steps of 6–10% while the position of the coil was constantly adapted until a pronounced foot lift upward was clearly visible without voluntary activation by the patient and without causing any pain or discomfort. The definite stimulation intensity was then individually adapted for each physical exercise to reach best level of muscular activation in the respective starting position (Table 2). During the training, the therapist followed the movements of the participant to continuously ensure the right coil positioning and effective stimulation during all repetitions of the exercises.

### 2.5. Outcome Measures

#### 2.5.1. Feasibility

Adherence: Adherence was defined as completing at least 9 of the 10 scheduled sessions within SPA and SPC, respectively. The reasons for the omission of sessions were documented. Safety: Participants completed customized questionnaires to document any adverse events (AEs) after every SPA and SPC session (Supplemental Figure S1). Prior to the start of each session, participants were asked by the therapist to report any AEs experienced between the sessions (Supplemental Figure S2). Practicability: During the training sessions, the physiotherapists documented all exercises with the respective positions and levels of difficulty as well as the stimulation parameters, including the definitive stimulation intensity, the number of repetitions performed, and the net stimulation time for each of the exercises. Further notes during and after the training sessions provided information about challenges and ideas for improvement of the setting and the intervention. Satisfaction: To assess the overall satisfaction with the intervention, customized questionnaires (semi-structured questions and open comment options) were completed after every second session by the participants (5 times during SPA and SPC, respectively) as well as at the end of the intervention by the participants and their caregivers (Supplemental Figure S1). At that time point, the participants and the caregivers were additionally asked about their motivation to repeat and recommend frNMS as a treatment option to other patients with similar conditions.

#### 2.5.2. Clinical Outcomes

At A1, A2, and A4, the participants and their caregivers completed the GOAL in its German paper-based version [57]. GOAL is a recently established patient-reported outcome measure. GOAL evaluates the gait priorities and functional mobility of children with cerebral palsy and is designed to assess all domains of the ICF-CY [57,58]. A change of ≥5 points in the total as well as the single domain scores were rated as an improvement given previously reported test–retest data [57]. GOAL data were obtained from 9 participants and 7 caregivers. The reasons for not filling in the questionnaire (i.e., missing data) were language difficulties and the length of the questionnaire. Therefore, GOAL was only obtained at time points A1, A2, and A4 but not A3 to enhance motivation to complete it.

In addition, a set of clinical parameters reflecting the ICF-CY domains “Body function and structure”, “Activity”, and “Participation” were assessed [59]. As no blueprint for this first-time frNMS study was available, the choice of clinical outcome parameters was met with regard to point-of-care measurements usually performed in clinical routines and only limited to clinical key parameters to not put too much additional strain on the study cohort. The strength of the ankle dorsiflexors was rated by the Medical Research Council (MRC) scale [51]. The Tardieu Scale was used to quantify the spasticity of the ankle plantar flexors by assessing the quality of the muscle reaction during a fast muscle stretch [60]. An increase in strength of ≥1 and a decrease in spasticity of ≥1 are regarded as clinically meaningful effects. The 10MWT was completed twice each at self-selected walking speed (SSWS) followed by maximum walking speed (MWS), respectively [61,62,63]. For the 10 MWT at MWS, the MDCs95 (minimal detectable change 95% confidence level) was previously reported as 1.7 s for GMFCS level I and 4.3 s and 17.7 s for GMFCS levels II and III, respectively [62]. For the clinical assessment, the examiner was not blinded to the study period.

### 2.6. Statistics

Due to the novelty of the frNMS protocol developed and applied targeting the tibialis anterior muscle by the research team for the first time, the study was primarily designed to assess its feasibility by means of adherence to the intervention. The adherence rate was calculated as the percentage of participants who did not discontinue the intervention. Accordingly, if any training sessions had been omitted for reasons related to the treatment itself (i.e., adverse events, discomfort, unwillingness to undergo the treatment), those interventions would have been categorized as not being adhered to. We predefined a threshold of completing at least 9 of the 10 a priori scheduled sessions as adherence to the intervention. Assuming that 90% of participants would adhere to the intervention, a sample size of *n* = 12 participants was intended to treat (CI ± 16.9). Given the qualitative nature of all other feasibility measures, no secondary endpoint sample size estimation was reasonable.

No a priori power analysis regarding the standardized clinical endpoints of the study could be performed, as this study reports the first experience applying the most recently developed frNMS protocol. No reliable data for such a longitudinal frNMS intervention are available to revert to.

All statistical analyses were performed using Microsoft Excel (Microsoft Office Professional Plus 2016, Microsoft Corp., Redmond, WA, USA) and SPSS (version 26/27; IBM SPSS Statistics for Windows, Armonk, NY, USA). Absolute and relative frequencies, means, standard deviations (SDs), medians, and ranges were calculated for treatment details, AE, and reports of satisfaction.

The feasibility of the frNMS intervention was descriptively explored on the basis of the adherence rate (see above), safety data, practicability as given by the adherence to the predefined stimulation and training protocol together with feedback from the therapists, and satisfaction with the intervention based on the feedback from the participants and their caregivers. Subjective, individual, and clinically meaningful effects were described through free-text comments by participants, caregivers, and therapists within the questionnaires.

Depending on the data distribution (tested by the Shapiro–Wilk Test), changes in GOAL scores over time were tested using Friedman tests and Benjamini–Hochberg false discovery rate correction. Changes in MRC and Tardieu scales were tested using Friedman tests. Changes in 10 MWTs were tested using repeated-measures ANOVAs. Bonferroni corrections were used for all post hoc comparisons. The level of significance was set to α = 0.05.

## 3. Results

### 3.1. Study Participants

Twelve participants (mean age 8.9 ± 1.6 years, five females (41.7%)) were enrolled in the study (Table 3).

### 3.2. Adherence

In SPA, 111 of 120 (92.50%) of the a priori scheduled training sessions were attended. In SPC, participants took part in 113 of 120 (94.17%) training sessions. In SPA and SPC, five and three participants omitted one session due to diagnostic measures interfering with the timing of the training sessions, respectively. In each of the study periods (SPA, SPC), two participants omitted two training sessions for interfering diagnostic measures. Altogether, 10 of the 12 participants (83%) took part in 9 out of 10 training sessions in both study periods. The a priori-defined primary adherence rate was 100%, as none of the training sessions were omitted due to adverse events, discomfort, or unwillingness to undergo the intervention.

### 3.3. Safety

In SPA (physiotherapy), AEs were reported only by one participant between sessions 3 and 4; the participant reported a tingling sensation in the lower leg. During SPC (frNMS), AEs were reported in 14 of 113 sessions, and no AEs were reported in 99 of 113 frNMS sessions (87.6%). One participant experienced pain twice during frNMS stimulation, which stopped immediately after pausing the stimulation. In addition, a tingling feeling was reported by five participants in eight sessions in total. Feelings of discomfort occurred in three sessions (muscle cramps reported twice by the same participant, warm sensation reported once). Stimulation was always paused until the discomfort resolved. Six participants did not report any AEs during the sessions. Pain at the stimulation site between sessions was reported by two participants once (6/10 or 5/10 on the visual analog scale). Pain spontaneously remitted before the start of the following session. A feeling of pressure was reported once by one participant after the first session. The caregivers of one participant reported a headache occurring once during SPC. Ten participants underwent SPC without reporting any AEs between sessions (see Table 4). None of the AEs led to the discontinuation of the frNMS intervention.

### 3.4. Practicability of frNMS

An average of 9460 ± 1054 stimuli per session were applied to the tibialis anterior muscle, while the average net stimulation time per session was 14.95 ± 1.63 min. The ten most frequently applied exercises are depicted in Table 1. From the therapist’s perspective, the intervention was rated as practical. Challenges included difficulties handling the machine while simultaneously ensuring treatment according to the protocol with correct coil positioning and constantly refocusing the participant and maintaining his/her motivation, particularly in younger children. Therefore, therapists experienced frNMS as more convenient to deliver if two therapists were present. Moreover, different levels of motor abilities, individual participants’ needs, and treatment goals required thorough preparation for each training session. Despite these challenges, frNMS was perceived as a helpful, promising approach by the therapists. From their perspective, frNMS very effectively counteracted the deficits in selective motor control, which is the cause of the unawareness of how to activate certain muscles. The scheduled duration of 45 min with 15 min net stimulation time for each session was rated as appropriate since the breaks, preparation for different exercises, and the child’s daily form needed to be accounted for during the training.

### 3.5. Satisfaction

Regarding SPA, 91.7% of participants and 75% of caregivers would repeat the physiotherapy. In addition, 83.3% of participants and 66.7% of caregivers would recommend the physiotherapy to other families with children with similar conditions. Regarding SPC, 91.7% of participants and 100% of caregivers would repeat the frNMS intervention. One participant stated that they would not repeat the intervention without giving a specific reason. Furthermore, 83.3% of participants and 91.7% of caregivers would recommend the frNMS intervention to other families with children with similar conditions. The reasons for not recommending frNMS were not given. According to the free-text comments, participants and caregivers particularly appreciated the child-friendly, customized setting for both SPA and SPC (Supplementary Table S1). General remarks pointed to the rather short intervention period with the desire for additional frNMS sessions to further foster the already achieved individual benefits. Patients and caregivers also often inquired about options to continue frNMS at home or during subsequent rehabilitation stays.

### 3.6. Participant/Caregiver-Reported Effects

**Figure 4:**
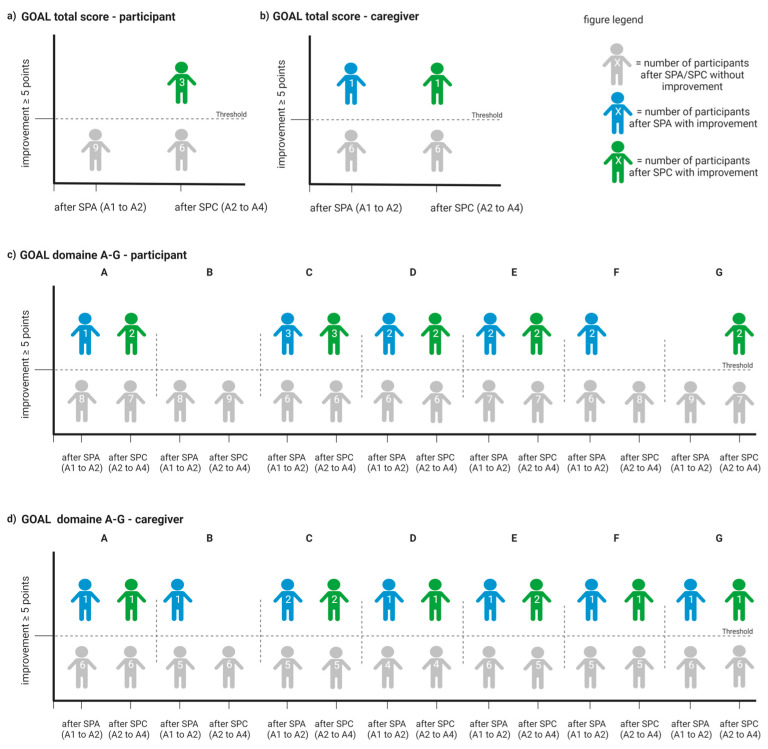
Infographic demonstrating improvements on the individual level for GOAL total score and domain scores A–G. (**a**) Report of GOAL total score completed by participants. (**b**) Report of GOAL total score completed by caregivers. (**c**) Report of domain scores A–G completed by participants. (**d**) Report of domain scores A–G completed by caregivers (created with BioRender.com).

On the individual level, GOAL improvements of ≥5 points compared to the respective previous assessment were observed 21 times in the domain reports completed by the participants (A2 to A1: n = 10 times, A4 to A2: n = 11 times; Table 5a and Figure 4). Regarding the caregivers’ reports, GOAL domain improvements of ≥5 points compared to the respective previous assessment were observed 16 times (A2 to A1: n = 8 times, A4 to A2: n = 8 times; Table 5b and Figure 4). On the group level, neither participant- nor caregiver-reported GOAL total scores or domain scores demonstrated significant changes over time (Table 5a,b).

### 3.7. Clinical Outcome Measures

**Figure 5:**
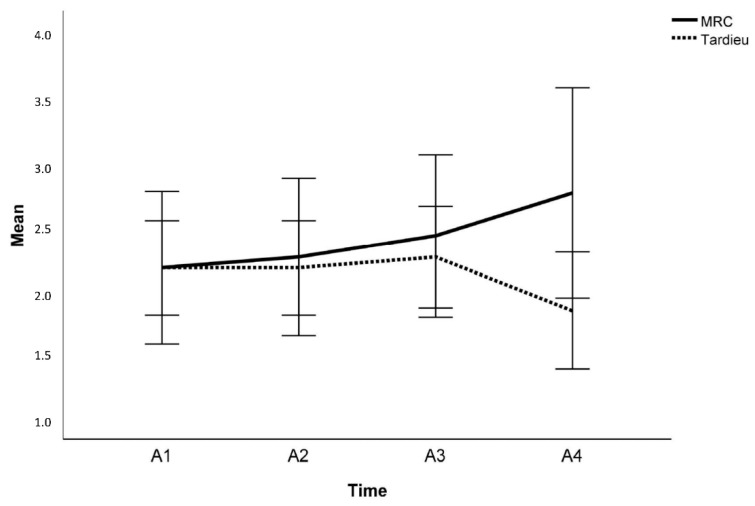
Group-level differences in mean medical research council (MRC) and Tardieu scores across the four assessments (A1–A4). MRC scores significantly differed across the four time points (Friedman test: χ^2^ = 12.40, *p* = 0.006). Mean MRC scores continuously increased from A1 to A4, albeit without statistical significance in the post hoc comparisons.

**Figure 6:**
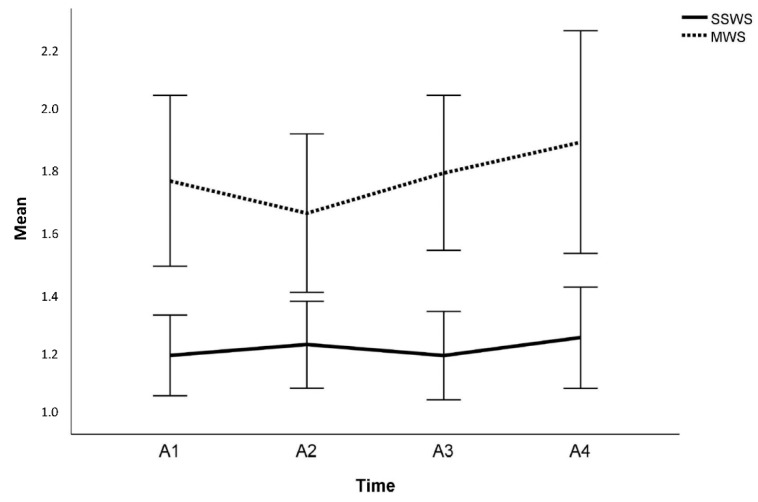
Differences in mean self-selected (SWSS) and maximal walking speed (MWS) in the 10 m walking test (10 MWT) across the four assessments (A1–A4).

On the individual level, MRC scores increased in five participants (after SPA in one participant; after SPC in four participants) and Tardieu scores decreased in four participants (no effect after SPA, but observed effects in four participants after SPC). For walking speed, no effects on the individual level were observed. On the group level, ankle strength measured by MRC significantly differed across the four time points (Friedman test: χ^2^ = 12.40, *p* = 0.006, Figure 4). Mean MRC scores continuously increased from A1 to A4, albeit without statistical significance in the post hoc comparisons (Table 6, Figure 5). The level of spasticity did not significantly change over the course of the study period (Table 6, Figure 5. Regarding the 10 MWT, no significant changes were observed for the SSWS or the MWS (Table 6, Figure 6).

## 4. Discussion

This feasibility study provides the first-ever data on the experience with a novel personalized frNMS intervention targeting the tibialis anterior muscle in 12 children affected by UMNS. The primary aim of this pilot study was to assess the feasibility of this most recently developed treatment approach by means of adherence to frNMS, safety and practicability of frNMS, and satisfaction with frNMS in combination with physiotherapy.

So far, rNMS has mainly been studied in adults affected by hemiparesis after stroke. The available evidence demonstrates beneficial effects with regard to muscle tone, strength, motor control, and pain in this cohort [20,39,48,49,54,64,65,66,67,68,69,70,71,72,73]. Because it is painless and does not require the attachment of electrodes or cables [20,21,25,41,74], rNMS represents an interesting option for pediatric patients, too. However, the pediatric evidence is limited to only one uncontrolled study and one case report enrolling a total of six children with spastic cerebral palsy so far. In these reports, benefits with regard to the range of motion of active and passive ankle dorsiflexion, the tone of the plantar flexors, and improved gait parameters (velocity, stride length, and cycle duration) were observed after five sessions of static rNMS targeting the tibial and peroneal nerves [48,49]. Against this background, feasibility studies and studies investigating the most effective treatment protocol in children are urgently needed to further explore this promising treatment approach in detail.

In this context, our research group developed a protocol for a functional rNMS intervention by combining electromagnetic stimulation and task-specific motor training targeting the tibialis anterior muscle. Since previous evidence has suggested active training to be superior to passive treatment modalities [16,75], a functional approach was chosen over static stimulation. As our frNMS intervention was primarily designed to enhance the strength and motor control of the targeted muscle, it does not comprise the stimulation of the (spastic) agonist of the respective limb(s). The stimulation protocol (frequency, alternating frequency, ON and OFF times, and total duration) was determined on the basis of previous reports of other research groups and our own experience [20,37,39,48,49,54,55,56,64,65,66,67,68,69,70,71,72,73,76,77,78,79,80,81,82].

In this study, adherence to the novel frNMS intervention in children affected by UMNS was as high as expected. For interventions in the field of motor rehabilitation, such high adherence rates are favorable and support that the patients accept the treatment very well. frNMS was as safe as conventional physiotherapy and as reported in pediatric treatment settings [48,49,83,84,85]. None of the few reported AEs led to the discontinuation or change of the treatment protocol. In terms of the practicability of the frNMS intervention, the protocol was conducted completely as planned with regard to the number of exercises performed and the total stimuli applied in almost all sessions. In this study, the time needed to define the optimal coil positioning and stimulation intensity had not been documented. From our latest everyday clinical experience with frNMS in our outpatient neuropediatric rehabilitation setting, we can report that this process in general takes 1.5 to 3 min. Yet, the therapists emphasized the multitasking ability needed to adhere to the protocol and keep the participant focused on the exercises. Therefore, currently, a setup with two therapists involved seems reasonable until technical progress eases the rNMS treatment. From the participants’ and caregivers’ perspective, satisfaction with the frNMS intervention was very high, as reflected by a high motivation to repeat and recommend the treatment [48,49,83,84,85]. To summarize, high adherence rates together with a high level of satisfaction with the intervention are very promising findings, as both criteria represent important considerations for establishing a new treatment approach in in- and outpatient rehabilitation centers.

The frNMS protocol was designed to deliver the neurostimulation in a very personalized way, realizing personalized medicine. Accordingly, the physical exercises and their level of demand were chosen depending on the participant’s individual priorities and capabilities. In this situation, participant-reported outcomes are particularly crucial to indicate the effects of the intervention. Specifically, GOAL and open-comment feedback were valuable instruments in our setup to specify benefits that were perceived as meaningful on the individual level. The open-comment feedback from the participants, caregivers, and therapists particularly emphasized positive changes in strength, motor control, and gait.

In addition, preliminary data on the effects regarding ankle dorsiflexor strength, plantar flexor spasticity, and walking speed were collected to evaluate their relevance as endpoints for future large-scale studies. On the individual level, clinically meaningful improvements were reported, as seen in the increased MRC scores in four participants after SPC (and in one participant after SPA) and the decreased Tardieu scores in four patients after SPC (no effect after SPA). No effects on the individual level were seen in the 10 MWT test. Given the limited sample size, this pilot study is likely to have been underpowered to reveal clinical benefits on the group level regarding these outcomes. In addition, the sequential study design without randomization of the treatment’s order could have biased the outcome. Despite introducing a 1-week pause between the study periods and separately comparing the pre and post measurements for each of the two study periods, a carryover from priming and/or training effects achieved during SPA to SPC cannot be definitely excluded. We decided against a cross-over design because we supposed that the carryover effects of frNMS would definitely bias a following physiotherapy intervention due to the strong proprioceptive activation by the neurostimulation. There was no possibility to enlarge the pause between study periods, as study participation was restricted to the duration of the inpatient rehabilitation stay (3 weeks). Furthermore, we decided against an inter-subject controlled design, as heterogeneity between children affected by UMNS also biases the interpretation of clinical findings in studies with small sample size. Again, the primary outcome was adherence rate, not the clinical effects. However, all these preliminary clinical findings will feed into the design of a future randomized trial.

Taking all of these promising real-world findings together, this feasibility study supports the need for future investigations of non-invasive neurostimulation from the bottom up by frNMS for children affected by UMNS. However, the small sample size and the non-sham controlled and non-blinded study design limit the generalizability of the observations at this stage. In addition, a specific statement about the effects on selective motor control cannot be made yet, as this has not been particularly addressed in this study or previous studies. However, frNMS has been postulated to exert its positive effects by enhancing selective motor control by promoting cortical and sensorimotor network reorganization. Therefore, an objective assessment tool to evaluate selective motor control (e.g., by the Selective Control Assessment of Lower Extremity (SCALE)), together with an objective and standardized assessment of the active range of motion and strength (e.g., by dynamometer), should be established in future trials [86,87,88]. Instrumented gait analysis to explore spatiotemporal parameters and changes in ankle kinematics, kinetics, and pattern of muscle activation could also add valuable information in this regard [89]. Furthermore, adding neurophysiological outcome measures (e.g., outcomes obtained by transcranial magnetic stimulation or functional magnetic resonance imaging) would contribute to the in-depth understanding of the mechanisms of action and enable even more personalized treatment protocols together with a stratification based on biologically sound response predictors.

In this study, the interventions took place during inpatient rehabilitation stays. The individually tailored selection of exercises for each participant and different combinations and levels of demand of exercise, as well as the different levels of attention during the training and different attitudes toward the intervention, may have contributed to differences in participant-reported outcomes on the individual level. Furthermore, this study does not provide insights into how long beneficial effects are sustained after frNMS, as no follow-up assessment took place. Flamand et al. (2014) reported the effects of the static treatment, which was delivered five times, lasted up to 45 days in their case report [49]. Consequently, future studies should include different follow-up time points to investigate the distinct clinical trajectory after the intervention. Different “dosages” (e.g., the time frame during which frNMS is delivered, the number of sessions, or the duration of a single session, including the number of exercises performed) should be explored as well.

## 5. Conclusions

The novel frNMS intervention designed to address ankle strength in children affected by UMNS turned out to be feasible with regard to adherence, safety, practicability, and satisfaction. Taking all these findings together, the approach can be considered very suitable for children with UMNS. However, frNMS requires further evaluation within large-scale, sham-controlled, randomized trials including clinical and neurophysiological outcomes (e.g., cortico-spinal excitability by transcranial magnetic stimulation) to provide information about the distinct mechanisms of action and the achievable clinical effects. Broader use of frNMS in the pediatric setting—even in the early stages of rehabilitation in intensive care units—may foster technical developments to further improve its applicability.

## Figures and Tables

**Table 1:** Description of the 16 exercises targeting to activation of the tibialis anterior muscle.

Name	Position	Description	Performed in *n* Sessions
Playing cards	Seated on a bench	Cards are placed under the tip of the foot; during stimulation, the patient lifts his foot, and 1 card is pulled out	39
Car race	Standing	Toy cars are placed on a knee-high ramp and held in place by the therapist, the patient’s foot is placed underneath the toy cars; during stimulation, the patient lifts his foot, tipping the toy car over the edge onto the ramp	35
Soccer	Standing	The patient’s foot is placed underneath a ball; during stimulation, the patient kicks the ball	34
Driving a car	Standing	A pedal is held down by the patient’s foot; during stimulation, the patient lifts his foot	32
Chicken rescue	Standing/seated	Small objects (e.g., toy birds) are balanced on top of the foot; during the stimulation, the patient moves his foot with the objects from one side to the other	27
Gym ball	Sitting on gymnastics ball	The patient rolls back and forth on the gymnastics ball and lifts his foot during stimulation	25
Stair-Climbing	Standing/walking	When stimulated, the patient climbs 1 step higher on the stairs	22
Rock climbing	Climbing	When stimulated, the patient climbs onto another climbing hold	18
Treadmill	Standing/walking	During stimulation, the patient performs a step in very slow motion while on a slow-running treadmill	17
Wobbly Surface	Standing on wobbly surface	Patient is standing on wobbly surface and lifts the foot when stimulated	16
Obstacle run	Standing/walking	When stimulated, the patient steps over an object (in slow or fast motion)	15
Side steps	Standing/walking	Patient focuses on performing a sidestep while actively lifting the foot when stimulated	13
Digger foot	Standing/seated	Patient grabs object with toes and, when stimulated, transports it to the hand or contralateral side	6
Cookie pricking	Standing/seated	Patient pricks cookies out of play dough by pressing down the cookie cutter with the foot; lifts the foot when stimulated.	4
Parallel bars	Standing/walking	Patient is walking while holding onto parallel bars, stimulation during gait phase pre-swing to initial contact during active dorsiflexion	3
Coloring	Standing/seated	Heel is dipped in paint; when stimulated, the patient paints something	1

**Table 2:** Definite stimulation intensities and their ranges in % of maximum stimulator output applied during the frNMS intervention (* for not to be specified reasons, patient 5 did not tolerate higher stimulation intensities during two exercises during the second frNMS session).

Pat.	Mean (%)	SD (%)	Minimum (%)	Maximum (%)
1	44.6	5.0	36	50
2	44.7	3.9	38	50
3	46.2	5.1	32	50
4	73.1	4.9	50	78
5	35.5	17.9	6 *	78
6	38.8	3.4	30	40
7	43.1	5.4	30	48
8	51.8	1.9	48	54
9	37.5	4.0	26	44
10	45.7	6.0	28	52
11	49.9	2.9	42	65
12	39.0	4.0	32	50

**Table 3:** Characteristics of the participants undergoing the frNMS intervention.

Pat.	Sex	Age *	Type of CP	GMFCS Level	(More) Affected Side	Etiology
1	F	7 y 7 m	USCP	I	Right	Perinatal stroke
2	M	8 y 0 m	USCP	I	Left	Perinatal stroke
3	M	6 y 7 m	USCP	I	Right	Intraventricular hemorrhage
4	F	8 y 5 m	BSCP	II	Left	Periventricular leukomalacia
5	M	10 y 2 m	USCP	I	Right	Hemorrhagic stroke
6	M	11 y 11 m	USCP	I	Right	Astrocytoma, completely resected
7	M	9 y 1 m	USCP	II	Left	Arterial ischemic stroke
8	M	10 y 11 m	USCP	I	Right	Perinatal stroke
9	F	7 y 11 m	USCP	I	Right	Periventricular gliosis
10	M	6 y 9 m	BSCP	II	Left	Periventricular leukomalacia
11	F	11 y 9 m	BSCP	II	Left	Periventricular leukomalacia
12	F	8 y 6 m	USCP	I	Right	Periventricular gliosis

* Age at baseline assessment. F, female; M, male; y, years; m, months; CP, cerebral palsy; USCP, unilateral spastic cerebral palsy; BSCP, bilateral spastic cerebral palsy; GMFCS, Gross Motor Function Classification System.

**Table 4:** Occurrence rate of adverse events (AEs) during the study.

AE	Occurrence Rate
**SPA: AE during sessions**	None
**SPA: AEs between sessions**	
Tingling sensation	0.8%
**SPC: AEs during sessions**	
Tingling sensation	7.1%
Feeling of local discomfort	2.7%
Pain	1.8%
Headache	0.9%
**SPC: AEs between sessions**	
Pain	1.8%
Pressure	0.9%

**Table 5:** (**a**) GOAL total and domain scores of participants at assessment A1, A2, and A4. (**b**) GOAL total and domain scores of caregivers at assessment A1, A2, and A4. Bold-printed change of ≥5 points compared with most recent previous assessment, meaning comparison between A1 and A2 and between A2 and A4; ADL = Activities of daily living, GFM = gait function and mobility, PDF = pain, discomfort, and fatigue, PASR = physical activities, sports, and recreation, GPA = gait pattern and appearance, UBMA = use of braces and mobility aids, BISE = body image and self-esteem; statistical tests used: Friedman tests, Benjamini–Hochberg false discovery rate correction. None of the significant *p*-values survived the Benjamini–Hochberg false discovery rate correction. Therefore, no post hoc testing was performed, as the other analyses did not show significant results.

	Total			A = ADL			B = GFM			C = PDF			D = PASR			E = GPA			F = UBMA			G = BISE		
Pat	A1	A2	A4	A1	A2	A4	A1	A2	A4	A1	A2	A4	A1	A2	A4	A1	A2	A4	A1	A2	A4	A1	A2	A4
(**a**)																								
**1**	83.3	82.5	84.7	97.5	87.7	**100**	100	98.0	100	81.6	83.7	**93.9**	75.0	**83.3**	83.3	66.7	**88.9**	80.6	75.0	50.0	50.0	62.5	45.8	37.5
**2**	--	--	--	--	--	--	--	--	--	--	--	--	--	--	--	--	--	--	--	--	--	--	--	--
**3**	69.5	65.5	**76.5**	95.1	95.1	97.5	93.0	--	91.0	59.2	**83.7**	**100**	39.6	**45.8**	50.0	75.0	77.8	80.6	25.0	0.0	0.0	45.8	25.0	**37.5**
**4**	57.7	59.7	**66.2**	71.6	**79.0**	79.0	72.0	72.0	76.0	73.5	73.5	**93.9**	35.4	37.5	**45.8**	41.7	38.9	**44.4**	25.0	**50.0**	50.0	45.8	45.8	50.0
**5**	91.4	91.4	**98.5**	93.1	93.1	**98.8**	96.0	96.0	99.0	98.0	98.0	98.0	81.3	81.3	**97.9**	91.7	91.7	**97.2**	--	--	--	87.5	87.5	**100**
**6**	--	--	--	--	--	--	--	--	--	--	--	--	--	--	--	--	--	--	--	--	--	--	--	--
**7**	58.2	58.4	58.4	55.6	56.8	56.8	76.0	76.0	76.0	95.2	95.2	95.2	43.8	43.8	43.8	44.4	44.4	44.4	25.0	25.0	25.0	33.3	33.3	33.3
**8**	83.5	83.4	84.1	91.7	92.1	93.7	88.8	88.8	88.8	93.9	93.9	93.9	86.7	86.7	86.7	77.8	77.8	80.6	100	100	100	54.2	54.2	54.2
**9**	62.9	64.2	62.9	80.2	80.2	80.2	76.0	76.0	76.0	79.6	83.7	77.6	50.0	50.0	50.0	52.8	**58.3**	55.6	0.0	0.0	0.0	33.3	33.3	33.3
**10**	64.2	66.4	65.3	86.4	86.4	86.4	75.0	74.0	74.0	63.3	**75.5**	71.4	--	--	--	52.8	55.6	52.8	0.0	0.0	0.0	45.8	45.8	45.8
**11**	--	--	--	--	--	--	--	--	--	--	--	--	--	--	--	--	--	--	--	--	--	--	--	--
**12**	66.0	68.6	68.6	69.1	69.1	69.1	78.8	82.5	82.5	91.8	**98.0**	98.0	40.5	42.9	42.9	75.0	75.0	75.0	25.0	**50.0**	50.0	41.7	41.7	41.7
**Mean**	70.7	71.1	73.9	82.3	82.2	84.6	84.0	82.9	84.8	81.8	**87.2**	91.3	56.5	58.9	62.6	64.2	67.6	67.9	34.4	34.4	34.4	50.0	45.8	48.1
**SD**	12.3	11.7	13.0	14.2	12.6	14.8	10.5	10.2	10.3	14.2	9.4	9.9	20.9	20.9	22.7	17.1	19.0	19.0	35.2	35.2	35.2	16.8	17.9	20.8
***Q***	6.94			8.40			2.80			5.55			8.59			5.36			< 0.001			1.71		
***p***	0.031			0.015			0.247			0.062			0.014			0.069			1.000			0.424		
(**b**)																								
**1**	63.1	**82.5**	84.4	80.2	**87.7**	**100**	82.0	**98.0**	100	71.4	**83.7**	**91.8**	52.1	**83.3**	83.3	55.6	**88.9**	80.6	25.0	**50.0**	50.0	25.0	**45.8**	37.5
**2**	--	--	--	--	--	--	--	--	--	--	--	--	--	--	--	--	--	--	--	--	--	--	--	--
**3**	73.8	70.5	**79.9**	92.6	95.1	96.3	96.0	90.0	91.0	81.6	83.7	**100**	42.9	40.5	**61.9**	80.6	77.8	**88.9**	25.0	0.0	**25.0**	--	25.0	**37.5**
**4**	56.5	57.1	58.5	72.8	72.8	72.8	65.0	63.0	66.0	79.6	79.6	79.6	37.5	41.7	45.8	36.1	38.9	38.9	50.0	50.0	50.0	37.5	37.5	37.5
**5**	85.7	85.7	86.2	88.9	88.9	88.9	91.1	91.1	91.1	95.9	95.9	95.9	77.1	77.1	79.2	91.7	91.7	93.3	--	--	--	66.7	66.7	66.7
**6**	--	--	--	--	--	--	--	--	--	--	--	--	--	--	--	--	--	--	--	--	--	--	--	--
**7**	56.4	55.7	56.0	49.4	49.4	50.6	--	--	--	97.6	97.6	97.6	50.0	50.0	50.0	52.8	52.8	52.8	50.0	50.0	50.0	37.5	37.5	37.5
**8**	74.1	74.1	75.7	84.1	84.1	85.7	85.7	85.7	85.7	91.8	91.8	91.8	--	--	--	63.9	63.9	**70.8**	75.0	75.0	75.0	45.8	45.8	45.8
**9**	--	--	--	--	--	--	--	--	--	--	--	--	--	--	--	--	--	--	--	--	--	--	--	--
**10**	64.2	66.4	65.3	86.4	86.4	86.4	75.0	74.0	74.0	63.3	**75.5**	71.4	--	--	--	52.8	55.6	52.8	0.0	0.0	0.0	45.8	45.8	45.8
**11**	--	--	--	--	--	--	--	--	--	--	--	--	--	--	--	--	--	--	--	--	--	--	--	--
**12**	--	--	--	--	--	--	--	--	--	--	--	--	--	--	--	--	--	--	--	--	--	--	--	--
**Mean**	67.7	70.3	72.3	79.2	80.6	83.0	82.5	83.6	84.6	83.0	86.8	89.7	51.9	58.5	64.0	61.9	67.1	68.3	37.5	37.5	41.7	43.1	43.1	44.0
**SD**	10.7	11.6	12.3	14.6	15.3	16.7	11.2	12.9	12.5	12.9	8.4	10.4	15.2	20.3	16.9	18.8	19.7	20.6	26.2	30.6	25.8	13.9	12.7	10.7
***Q***	5.85			7.43			2.53			4.67			5.57			4.30			1.00			2.00		
***p***	0.054			0.024			0.282			0.097			0.062			0.116			0.607			0.368		

**Table 6:** Change of clinical measures assessed for the (more) affected lower limb induced by physiotherapy and frNMS at assessments 1, 2, 3, and 4 (A1–A4). Medical Research Council (MRC) scale for assessing the power of ankle dorsiflexion; bold printed = increase of ≥1.0, which was regarded as a substantial meaningful change on the individual level; Tardieu Scale (Tardieu) as a measure of spasticity of the plantar flexors, bold printed = decrease of ≥1, which was regarded as a substantial meaningful change on the individual level; self-selected (SSWS) and maximum walking speed (MWS) in 10 m walking test (10 MWT). Statistical tests used: Friedman tests, repeated-measures ANOVA, Dunn–Bonferroni post hoc tests. Significant differences are marked with an asterisk (*). Only for MRC, the post hoc testing was performed, as the other analyses did not show significant results.

	MRC				Tardieu				10 MWT							
									SSWS (m/s)				MWS (m/s)			
Participant	A1	A2	A3	A4	A1	A2	A3	A4	A1	A2	A3	A4	A1	A2	A3	A4
**1**	2	2	3	3	2	2	2	2	1.10	1.09	0.76	1.00	1.85	1.69	2.10	2.01
**2**	0	0	0	0	1	1	1	1	1.17	1.10	1.25	1.15	1.80	2.01	2.34	2.81
**3**	2	**3**	3	**4**	3	3	3	**2**	1.15	1.16	1.31	1.18	1.90	1.89	2.10	2.04
**4**	2	2	2	2	3	3	3	**2**	1.13	0.94	1.22	1.07	2.75	2.29	1.83	1.97
**5**	3	3	3	**4**	2	2	2	**0**	1.18	1.17	1.30	1.28	1.72	1.78	1.80	1.83
**6**	3	3	3	3	2	2	2	2	1.33	1.48	1.27	1.35	2.03	1.83	2.25	2.68
**7**	1	1	1	1	2	2	2	2	0.83	0.96	0.85	1.00	1.76	1.08	1.34	1.16
**8**	2	2	3	**4**	2	2	2	2	1.34	1.09	1.11	1.37	2.00	1.80	1.88	2.25
**9**	3	3	3	**4**	2	2	2	2	1.64	1.69	1.58	1.81	1.60	1.60	1.68	1.79
**10**	3	3	3	3	2	2	2	2	1.23	1.25	1.21	1.15	1.35	1.16	1.29	1.19
**11**	2	2	2	2	3	3	3	**2**	0.91	1.25	1.00	0.97	0.97	0.90	1.04	0.94
**12**	3	3	3	3	2	2	3	3	1.22	1.48	1.39	1.63	1.36	1.79	1.75	1.93
**Mean**	2.17	2.25	2.42	2.75	2.17	2.17	2.25	1.83	1.19	1.23	1.20	1.26	1.75	1.64	1.77	1.87
**SD**	0.94	0.97	1.00	1.29	0.58	0.58	0.62	0.72	0.21	0.24	0.24	0.28	0.44	0.40	0.40	0.57
***Q/F***	*Q* = 12.395				*Q* = 6.750				*F* = 0.759				*F* = 1.841			
***p***	0.006 *				0.080				0.525				0.191			
***p^A1–A2^***	1.000															
***p^A1–A3^***	1.000															
***p^A1–A4^***	0.414															
***p^A2–A3^***	1.000															
***p^A2–A4^***	0.683															
***p^A3–A4^***	1.000															

## Data Availability

The data presented in this study are available on request from the corresponding author. The data are not publicly available due to the sensitive character of pediatric clinical data.

## Supplementary Materials

The following supporting information can be downloaded at: https://www.mdpi.com/article/10.3390/children10101584/s1, Supplemental Table S1: Feedback given by therapists, participants, and caregivers during the study period. All pronouns are replaced by “child” to ensure anonymity. Abbreviations: CG = caregiver, Pat. = participant, T: therapist; Supplemental Figure S1: Questionnaires for participants and their caregivers used to assess the satisfaction with the frNMS treatment; Supplemental Figure S2: Treatment documentation questionnaire, completed prior to and after every session to assess any adverse events occurring during or after treatment sessions.

## Abbreviations

A1	Assessments including clinical measures and participant-reported outcomes completed before SPA
A2	Assessments including clinical measures and participant-reported outcomes completed after SPA
A3	Assessments including clinical measures and participant-reported outcomes completed before SPC
A4	Assessments including clinical measures and participant-reported outcomes completed after SPC
AE	Adverse events
CP	Cerebral palsy
GOAL	Gait Outcomes Assessment List
ICF-CY	International Classification of Functioning, Disability and Health-Children and Youth Version
MDCs95	Minimal detectable change 95% confidence level
MRC	Medical Research Council
MWS	Maximum walking speed
(f) rNMS	(Functional) repetitive neuromuscular magnetic stimulation
SCALE	Selective Control Assessment of Lower Extremity
SD	Standard deviation
SPA	Study period A
SPC	Study period C
SSWS	Self-selected walking speed
UMNS	Upper motor neuron syndrome
10 MWT	10 Meter Walking Test
