# Supplementary material for: Functional Repetitive Neuromuscular Magnetic Stimulation (frNMS) Targeting the Tibialis Anterior Muscle in Children with Upper Motor Neuron Syndrome: A Feasibility Study
Source: Children (Basel). 2023 Sep 22;10(10):1584. doi: 10.3390/children10101584 (PMC10605892; doi:10.3390/children10101584)
Supplement: Supplementary file 1 [file children-10-01584-s001.zip › children-2563644-supplementary.pdf]

Supplemental Figure S1: Questionnaires for participants and their caregivers used to assess the satisfaction with the frNMS treatment

Patient's questionnaire after every second session

1. This questionnaire was filled in by:

    1 me

    1 my parent/a third person

2. This questionnaire was filled in on: ______________________

3. Were you positioned comfortably during the session?

1 Yes

1 No, because ____

____

4. How did the treatment feel?

____

____

5. Did you experience any pain or discomfort during the session?

1 Yes; please specifiy the severity of the pain using the emoticons below and let us know, where you experienced pain:

____

____

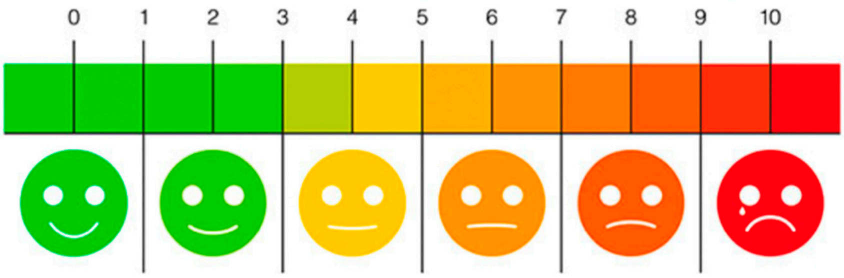

☐ No

6. Would you repeat this treatment?

1 Yes

1 No, because… ____

____

7. Does the treated body part feel different now?

1 Yes, how?

____

____

1 No

8. Does any other body part feel different now (sensation of heat, itching, burning)?

1 Yes, where? ____

____

1 No

9. Further comments:

____

____

____

____

Thank you very much for answering these questions and for your feedback!

Questionnaire for patients after the treatment with rNMS

Study-ID: ___________________________________

1. This questionnaire was filled in by:

    1 me ☐ caregiver/a third person

2. This questionnaire was filled in on: ____________________

3. Overall, how did you experience the treatment?

____

____

4. Would you undergo the treatment again?

1 Yes

1 No, because ____

____

5. Does the treated body region feel different in comparison to before the intervention?

1 Yes, how?

____

____

1 No

6. Does any other body region feel different in comparison to before the intervention?

1 Yes, where?

____

____

1 No

7. Would you recommend this treatment method to other children/adolescents with movement restrictions?

1 Yes

1 No, because ____

____

8. Could/Should we have done anything differently during the treatment?

1 Yes, what?

____

____

1 No

10. Further comments:

____

____

____

____

Thank you for completing the questionnaire!

Questionnaire for caregivers at the end of the patients´ rNMS treatment

Study-ID: ____

1. This questionnaire was filled in by: 1 Mother 1 Father

2. This questionnaire was filled in on: ____________________

3. Do you notice a difference in your childs behavior in comparision to before the treatment?

   1 No
   1 Yes, which?

   ____

   ____

4. Did your child comment on how it liked the treatment?
   1 No
   1 Yes, how?

   ____

   ____

5. Did your child complain about pain or other sufferings (headache, pain in legs, bottom or hip etc.)?

   1 No
   1 Yes, where and what?

   ____

   ____

6. Would you let your child undergo the treatment again?
   1 Yes
   1 No, because ____

   ____

7. Would you recommend this therapy to other familys with children with movement restrictions?

   1 Yes
   1 No, because____

8. Do you have any ideas to improve the process?
   1 No

   1 Yes, which?

   ____

   ____

9. Further comments:

____

____

____

____

Thank you for completing the questionnaire!

Supplemental Figure S2: Treatment documentation questionnaire, completed prior and after every session assess any adverse events occurring during or after treatment session

frNMS Treatment Documentation

Study-ID: ______________________

Session Nr.: ____________

Date: __________________________

1. Did the last rNMS session cause any adverse events? If so, …

… which did you notice?

€ Pain
€ Feelings of Pressure
€ Muscle Sore
€ Muscle Tremor
€ Tingle
€ Numbness
€ Cold Feelings
€ Warm Feelings
€ Burning Sensation
€ Furry Feelings
€ Other Adverse Events: ____

€ No

… when did they occur?

€ In Motion
€ While Resting
€ Constantly/Always

… how strong where they (VAS 0-10)? __________________________________

… how long did they last? ____

2. Did the patient feel pain in hip or legs before the beginning of the session?

- € No
- € Yes

3. Did the patient report any adverse events or unpleasant sensations during the rNMS session? If so, …
… which occured?

- € Pain
- € Feelings of Pressure
- € Muscle Sore
- € Muscle Tremor
- € Tingle
- € Numbness
- € Cold Feelings
- € Warm Feelings
- € Burning Sensation
- € Furry Feelings
- € Other Adverse Events: ____

- € No

Supplemental Table S1: **Feedback given by therapists, patients and caregivers during the study period.** Any pronouns were replaced by "child" to ensure anonymity. Abbreviations: CG = Caregiver, Pat.=patient, T: Therapist

**Participant**	**Feedback by whom and when**	**Comment**
**1**	T after SPC	Pronounced improvement of motor control, active movement execution visible
	CG after SPC	More sessions would be desired to better see positive results
**2**	CG after SPA	Very child-friendly setting
**3**	Pat after session 5 & 8 SPA	My leg feels different – in a good way
	CG after SPA	Child is looking forward to every session.
	Pat during SPC	Very cool!
	CG after SPC	Child was excited for every session. Amazing intervention, we would repeat it any time.
**4**	CG after SPA	Child had a lot of fun.
	Pat during SPC about frNMS	This was really cool, I want to do it again.
	CG after SPC	Child enjoyed the treatment a lot.
**5**	CG after SPA	The treatment was received well by child.
	Pat after session 2 SPC	Lifting my foot is easier now.

	CG after SPC	Child liked all the sessions
	Pat after SPC	I can lift my toes better.
**6**	Pat during SPA	I feel more tired in general.
	CG after SPA	Child liked it.
	Pat after session 6, SPC	The treatment feels good.
	CG after SPC	Child liked it a lot, it was fun for him, sometimes strenuous, but child likes to do stuff like that!
**7**	CG after SPA	Child enjoyed the treatment and spoke positively about it.
	Pat after session 2 SPC	My foot feels cool
	Pat after SPC	My foot is a lot better now, but can't exactly say, in what way.
	CG after SPC	Child is able to better and easier lift the foot. In the beginning [of frNMS] child was fascinated and thrilled, in the end, it was little much with the other therapies.
**9**	Pat during SPA	More momentum upwards and more strength when I'm pressing down my foot. I enjoy this a lot! Walking is better, my foot feels way better than usual.
	CG after SPA	Child was looking forward to every session all day, child spoke very positively, and the foot got a little better every day. Child could touch the foot down without orthoses more controlled after the first days.
	Pat during and after SPC	This feels really good and very nice. It helps my foot; I can walk better/more beautifully now. Maybe sometimes, we could take a break with the stimulation.
	CG after SPC	Child's gait worsened a little bit when not wearing orthoses, but child wasn't wearing orthoses at all during this week, so maybe it's due to exhaustion. Child enjoyed it a lot and was always very motivated.
**10**	Pat after SPA	Walking on my heels works better now.
	CG after SPA	Child is able to walk better and longer. More endurance, less tripping! Child likes the therapist a lot, talks a lot about the treatment and enjoy it.
	CG after SPC	In the beginning, child liked it lot, towards the end he was often tired, there were a lot of other therapies as well.
**11**	Pat after SPA	My foot/leg feels stronger now.
	Pat during and after SPC	The muscle feels more at ease, it's easier to move. My foot feels more relaxed. It feels much better.
	CG after SPC	Child's foot is more relaxed, not as tense.
**12**	Pat during and after SPA	My foot feels better, I have more power when playing soccer.
	CG after SPA	Interesting, fun, challenging (in a good way) according to child.

	Pat after SPC.	frNMS feels cool, my foot and lower leg feel better, but I can’t describe how. My foot doesn’t feel smaller than the other one anymore.
	T during SPC	Throughout frNMS-period, it got easier to find the right location to stimulate, muscle contracts more pronounced, desired movement is executed by patient more pronounced and smoother.
	CG after SPC	Climbing stairs has improved. Child was thrilled and motivated every day.
